# Artificial capillaries-on-a-chip with modular control over lumen size, architecture, and co-culture conditions

**DOI:** 10.1088/1758-5090/ae7834

**Published:** 2026-06-18

**Authors:** Arun Poudel, Joshua Edwin Nana Limjuico, Ujjwal Aryal, Md Tariqul Hossain, Saikat Basu, Pranav Soman

**Affiliations:** 1 Department of Biomedical and Chemical Engineering, Syracuse University, Syracuse, NY, 13244, United States of America; 2 Department of Mechanical Engineering, South Dakota State University, Brookings, SD 57007, United States of America

**Keywords:** artificial capillaries, femtosecond laser cavitation, microfluidic chip, top-open silos, co-culture

## Abstract

Currently, *in vitro* models of microvascular biology rely on self-assembly of vascular cells in compatible gels. However, the stochastic nature of this process results in large variations in lumen sizes, perfusion continuity, and shear stresses, making systematic and reproducible analysis challenging. Here, we report a new technology to generate artificial capillaries on a chip with custom control over lumen sizes and architectures using a combination of femtosecond laser cavitation and collagen casting within multi-chambered microfluidic chips. The design allows seeding of endothelial cells within capillary-sized microchannels and seeding of stromal cells within top-open silos, with independent control over seeding sequence and media compositions. Results show that endothelialized microchannels, coined as artificial capillaries, exhibit excellent barrier function with reproducible control over lumen sizes (*φ* = 8–40 *µ*m) and their architectures (straight, curvatures, tapered, branched). The physical flow parameters measured across the lumen (namely, flow shear) and at the channel outlets (flow velocities) have been validated against high-fidelity numerical assessments from the Large Eddy Simulation scheme within the digitized versions of microchannels. The experiment-computation compatibility enabled us to predict changes in regional velocity and wall shear stresses within artificial capillaries for various capillary architectures. We also show that *in situ* editing of artificial capillaries in the form of adding new branches or adding occlusions is possible. Lastly, we developed a co-culture model that enables the study of stromal cells with artificial capillaries using conventional imaging methods. We envision that acellular chips with two seeding ports can be readily shipped worldwide and could potentially be adopted as a new technology to study microvascular biology in a reproducible manner.

## 1. Introduction

Since capillaries are the primary site of nutrient exchange between tissue and blood circulation, understanding the mechanisms regulating capillary function in health and disease can be beneficial for the development of new therapies. Inspired by angiogenesis and vasculogenesis processes, many *in vitro* models have been developed to study microvascular biology. Such models typically involve the use of endothelial cells within fibrin gels, forming networks via self-assembly. However, the stochastic nature of capillary formation results in poor reproducibility with a wide range of lumen sizes (5–200 *μ*m), unpredictable perfusion connectivity, and locally varying fluid flow and shear stresses, which make a systematic study about microvasculature challenging [1–5]. To increase reproducibility, molding, bioprinting, and laser processing have been widely used to pre-defined endothelialized channels [6–20]. The simplest method involves the removal of a tubular mold from polydimethysiloxane (PDMS) chips to leave behind a lumen (∼150–550 *μ*m) embedded within ECM matrix, which is then lined with endothelial cells. To go beyond planar topology, 3D bioprinting has also been used, although each method enforces strict constraints on bioink composition. For instance, extrusion-based direct printing requires high-viscosity bioinks and rapid crosslinking mechanisms to print robust structures, while indirect printing requires the use of sacrificial materials that can be removed without generating any cytotoxic side effects, and light-based printing requires photo-sensitive and low-viscosity bioinks. At present, only multiphoton laser ablation or degradation can generate capillary-sized channels [21–25], although negative impact on cell viability during laser scanning and endothelializing channels less than 50 *μ*m lumen size remains difficult. To address these challenges, we report a new technology to generate highly reproducible artificial capillaries embedded within unmodified extracellular matrix (collagen). Here, we have user-defined control over lumen size (8–40 *µ*m), architectures (branching, tapers, zig-zag), *in situ* editing, and incorporating stromal cells during active cultures, yet enable easy-to-use cell seeding method, and be compatible with standard imaging and analysis methods.

## 2. Results

### 2.1. Design and fabrication of chips to generate capillary-sized microchannels in 3D collagen

**Figure 1.**
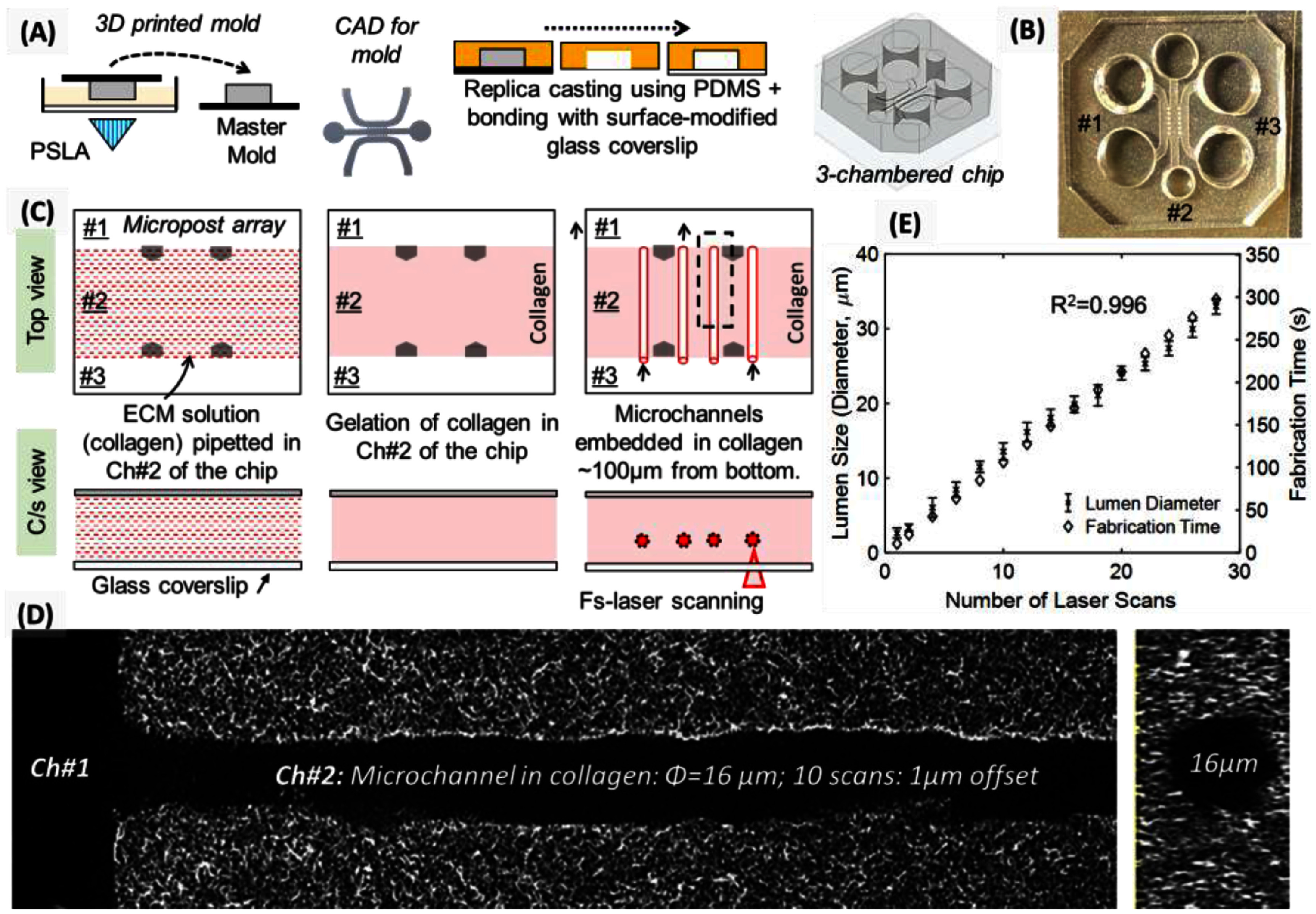
(A) Process flow showing the fabrication process to make PEGDA master molds and final PDMS device for chip design A. (B) CAD model of a three-chambered chip and a representative photograph of final PDMS chip bonded to glass coverslip. Photograph shows chamber 1–3 (Ch#1, Ch#2, Ch#3) separated by an array of microposts (C) Top and cross-sectional schematic views highlighting the process flow to make endothelialized microchannels within collagen (artificial capillaries) inside chips. (D) Confocal reflectance microscopy images of top and cross-sectional views of a representative microchannel embedded in collagen matrix. (E) Plot showing the relationships between target lumen size of microchannels, the number of laser scans, and total fabrication durations. Linear regression revealed a strong correlation (*R*^2^=0.996).

Briefly, a combination of 3D printed master molds, collagen casting, and femtosecond laser-assisted cavitation was used to generate capillary-sized microchannels embedded within collagen within PDMS chips. First, projection stereolithography (PSLA) was used to make customized three-chambered microfluidic devices using PDMS microfluidic devices. Here, digital light patterns (405 nm) modulated by digital micro-mirror devices was used to print a negative master mold using a photosensitive PEGDA (250kDa) resin (figures 1(A) and SI-1). Replica molding was used to generate PDMS chips with three chambers separated by an array of microposts (figures 1(B) and SI-2). The total height of the chip was 4 mm, while the height for all three chambers in the chip was 200 *µ*m. The final devices consist of three chambers: a central chamber (Ch#2; 0.9 mm wide, designed to house crosslinked collagen) flanked on either side by two side chambers (Ch#1 and Ch#3; 1 mm wide, for media exchanges). In this work, we choose type I collagen as a model ECM due to its abundance in native tissues and its wide use for developing 3D cell culture models. Before pipetting collagen solution within PDMS chips, the chips were surface modified to prevent delamination of crosslinked collagen from the channel surfaces during active culture. Then, type I collagen solution (5 mg ml^−1^) was gelled within the central chambers of UV-sterilized chips. After 24 h, a focused femtosecond laser (1.3 W, 40X water immersion objective, 0.8 N.A., 800 nm) was used to generate 3D microchannel architectures. To reliably generate 3D channels of defined lumen sizes, laser scanning was performed within collagen gel about 50 *µ*m above the bottom glass surface (figure 1(C)). Reflectance microscopy images show top and cross-sectional views of microchannels embedded in collagen matrix (figures 1(D) and SI-3) Laser dosage above 2 × 10^5^ Jcm^−2^ resulted in cavitation and formation of lumens of ∼3 *µ*m size. During laser scanning, the radial expansion of the bubble generates a shockwave that locally breaks down the collagen network and leaves behind a hollow lumen in its wake. Protocols were optimized to ensure repeatable and stable channel formation without collapse. At this speed, a single scan of 1 mm takes ∼10 s. In a single scan, a lumen size of 3 *µ*m can be generated while multiple scans with varying lateral offsets (1, 2, and 4 *µ*m) were optimized to generate lumen size from ∼3–40 *µ*m (SI-3). Based on the fabrication speed of 100 *µ*m s^−1^, a lumen of 20 *µ*m required ∼16 scans, with a duration of 170 s while a lumen size of 35 *µ*m required ∼28 scans, corresponding to around 298 s of fabrication time. The plot (figure 1(E)) shows the relationship between the number of laser scans, target lumen size, and total fabrication time. For this plot, we used 3 independent chips with 5 measurements per chip for each channel size. Lumen sizes, as measured by confocal reflectance microscopy across multiple chips, showed a high degree of reproducibility with a nearly circular cross-section. These laser-sculpted chips could be stored under hydrated conditions (PBS in side-chambers) for up to 3 months without any obvious effect of deterioration; however, all chips used for cell-culture experiments were performed within 2 weeks of fabrication.

### 2.2. Optimization of perfusion culture in chips (figure 2)

**Figure 2.**
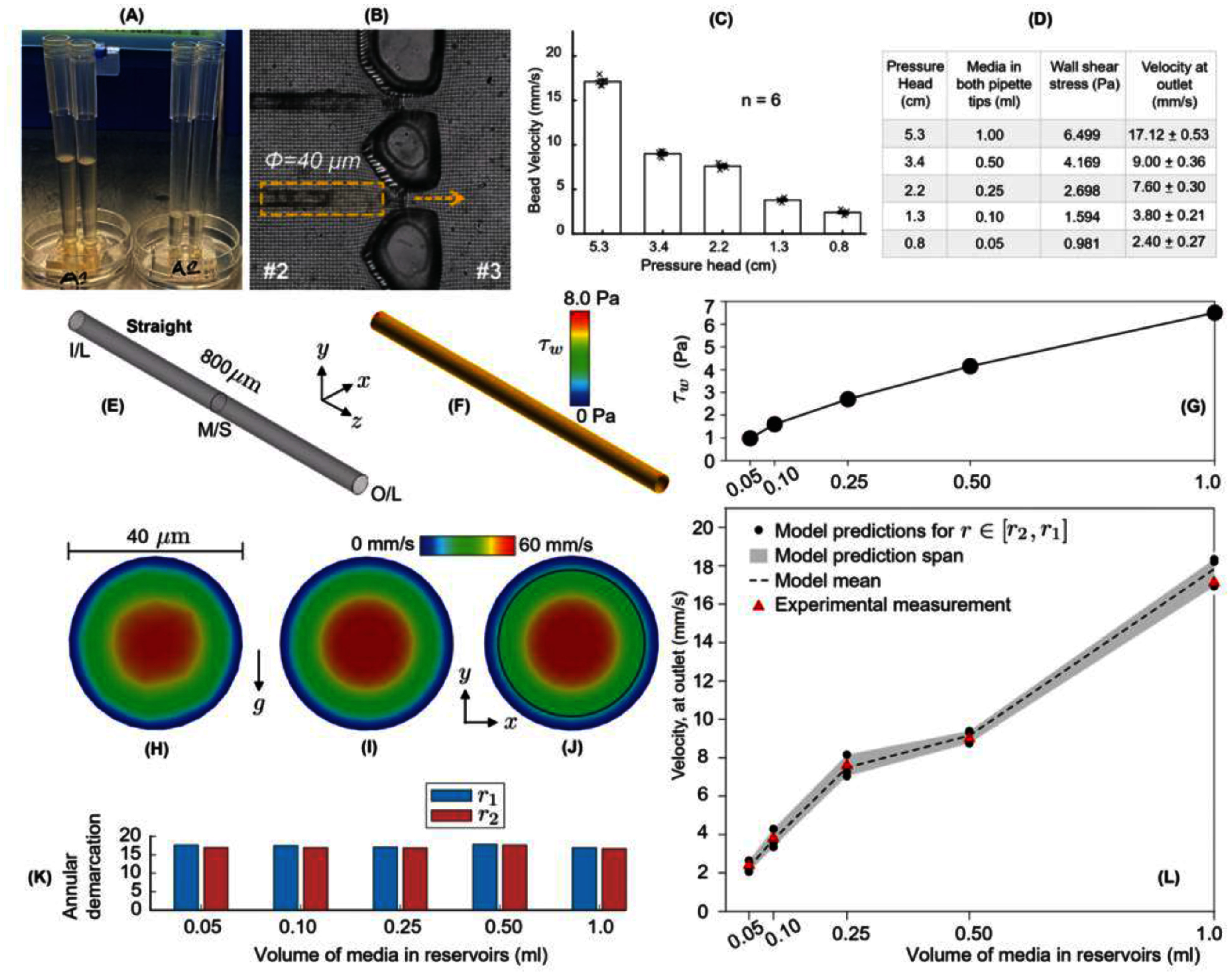
(A) Photograph of hydrostatic pressure driven perfusion setup showing 1 ml pipette tips inserted in the inlet and outlet ports of Ch#1 while other ports are open to atmospheric pressures; this enables directional flow of media from Ch#1 to Ch#3 through microchannels embedded within collagen in Ch#2. (B) brightfield image showing two microchannels (lumen size = 40 μm) at the interface of Ch#2 and Ch#3 while dashed box highlights the region of interest (ROI) used to calculate the velocity of microbeads. (C) Plot and table shows the relationships between pressure-head, bead-velocities, and calculated wall shear stresses generated within the microchannels. (E). Digitized straight microchannel, showing the identified locations for inlet (I/L), mid-point section (M/S), and the outlet (O/L). (F). Surface map of the area-weighted average wall shear with the simulated pressure head at 5.3cm. (G). Simulated wall shear values, for the tested range of pressure heads (labeled in terms of the volume of media in the pressure head reservoirs). (H)–(J). Simulated velocity fields, respectively at the inlet, mid-point, and outlet of the digitized microchannel (per panel (E). (K) Data on the annular region location in the outlet field, bounded by *r*_1_ and *r*_2_ , commensurate with the experimentally measured bead velocities. (L) Experiment-computation comparison for velocity measurements at the microchannel outlet for the tested pressure heads.

Gravity-based flow was used to generate perfusion within microchannels embedded in collagen. To induce unidirectional flow, 1 ml pipette tips were fitted to the inlet and outlet ports of chamber 1, and both pipettes were filled with equal volumes of PBS (or media during active cell-cultures) while the inlet and outlet ports of chamber 3 were left open (figure 2(A)). Hydrostatic pressure gradient across the collagen gel, corresponding to the PBS height in pipettes, initiated a range of velocities in microchannels. To calculate the velocities within the microchannels, chips with 6 microchannels of lumen size 40 *µ*m were developed. Different volumes (0.05–1 ml) of microbead solution (yellow-6.0 *µ*m) were filled in the pipette tips, and time-lapse movies of bead movement were recorded (Hamamatsu, 40 fps). A region of interest, inside the lumen in chamber 2 was identified (figure 2(B)), dashed yellow box), and velocity measurements were averaged across 4 independent chips with six measurements for each channel size. Bar plot shows that bead velocity decreased with a decrease in pressure head (figure 2(C)). The table shows that by simply changing the media volume, we can achieve a shear stress range between ∼10 and 65 dynes cm^−2^ (figure 2(D)) close to the *in vivo* range reported in the literature (5–95 dynes cm^−2^).

Due to scattering induced by the collagen matrix, it was challenging to measure bead velocities for smaller lumen sizes, so we developed new computational models to calculate the velocities and wall shear stresses. We also numerically simulated the pressure gradient-driven flow physics within a digitized replica of an isolated straight microchannel (figure 2(E)), spatially segregated into approximately 1.7 million graded, unstructured tetrahedral elements. The mesh resolution within the in silico domain was carefully derived from a grid sensitivity analysis of the simulated flow outcomes (see SI-4). For reliable computational fluid dynamics (CFD) estimation of the transport parameters, we implemented the high-fidelity large eddy simulation (LES) scheme, with the dynamic kinetic energy transport model resolving the subgrid-scale fluctuations (the simulation time-steps were 0.0001 s; see [26] for related mathematical formalism applied in a different micro-scale physiological system). The ambient pressure conditions in the simulations were guided by the corresponding experimental data (so, separate simulations were developed with pressure heads of 5.3, 3.4, 2.2, 1.3, and 0.8 cm—applied from inlet I/L to outlet O/L; see figure 2(E)). The simulated area-weighted average wall shear for the 5.3-cm pressure head was 6.45 Pa; the surface map is shown representatively in figure 2(F). We have subsequently plotted all the simulated wall shear averages for the different pressure head conditions (see figure 2(G)); the specific values are *τ_w_*** =**6.45, 4.15, 2.69, 1.59, and 0.98 Pa—respectively for the highest-to-lowest pressure heads. The trend matches the experimental assessments for shear stresses (based on the measured velocity gradients); see the table in panel 2D. The simulated velocity fields are shown in figures 2(H)–(J); therein note the narrow black annular region in the outlet field in figure 2(J). The experimental data (see table) indicated bead velocities of *v_e_* = 17.12, 9.0, 7.6, 3.8, and 2.4 mm s^−1^ at the microchannel outlet (respectively under pressure heads of 5.3, 3.4, 2.2, 1.3, and 0.8 cm). The corresponding standard deviations on the velocities were *σ*= 0.53, 0.36, 0.30, 0.21, and 0.27 mm s^−1^. From the Hagen–Poiseuille equation, we estimated the annular region extents in the simulated outlet field (e.g. in figure 2(J)) where the velocities stay within *v_e_* ± *σ*; for instance, for the 5.3 cm pressure head, the annulus is bound by *r*_1_ = 17.65 mm and *r*_2_ = 16.59 mm (the comprehensive set of {*r*_1,_ *r*_2_} all pressure gradients is shown in figure 2(K)). The finding conforms with the expectation for the bead locations to be skewed toward the channel walls during the experimental measurements (owing to the diverging nature of the flow streamlines at the exit cross-section). From the numerical grid points within this annulus, we extracted the simulated velocity values; for the 1.7 million element mesh, there were three such grid points within the narrow annulus. The validative comparison between the simulation-derived channel outlet velocities and the experimental measurements (with red markers) is demonstrated in figure 2(l). In context of the computational simulations of intra-capillary transport, the reproducibility assessment focused on the numerical pipeline rather than on independent fabrication runs. Specifically, we verified mesh sensitivity by confirming that the flow outcomes exhibit asymptotic convergence across successively refined mesh resolutions (e.g. see SI-4), indicating that the reported quantities are not artifacts of a particular discretization. We additionally confirmed numerical stability by ensuring that the Courant number (a dimensionless number evaluating numerical stability of the in-silico models and is the ratio of the physical distance a particle moves during a single time step to the spatial grid spacing) remained below unity. Together, these checks support the reproducibility of the reported results with respect to the discretization and solver configuration.

### 2.3. Endothelialization of capillary-sized channels embedded within collagen matrix

**Figure 3.**
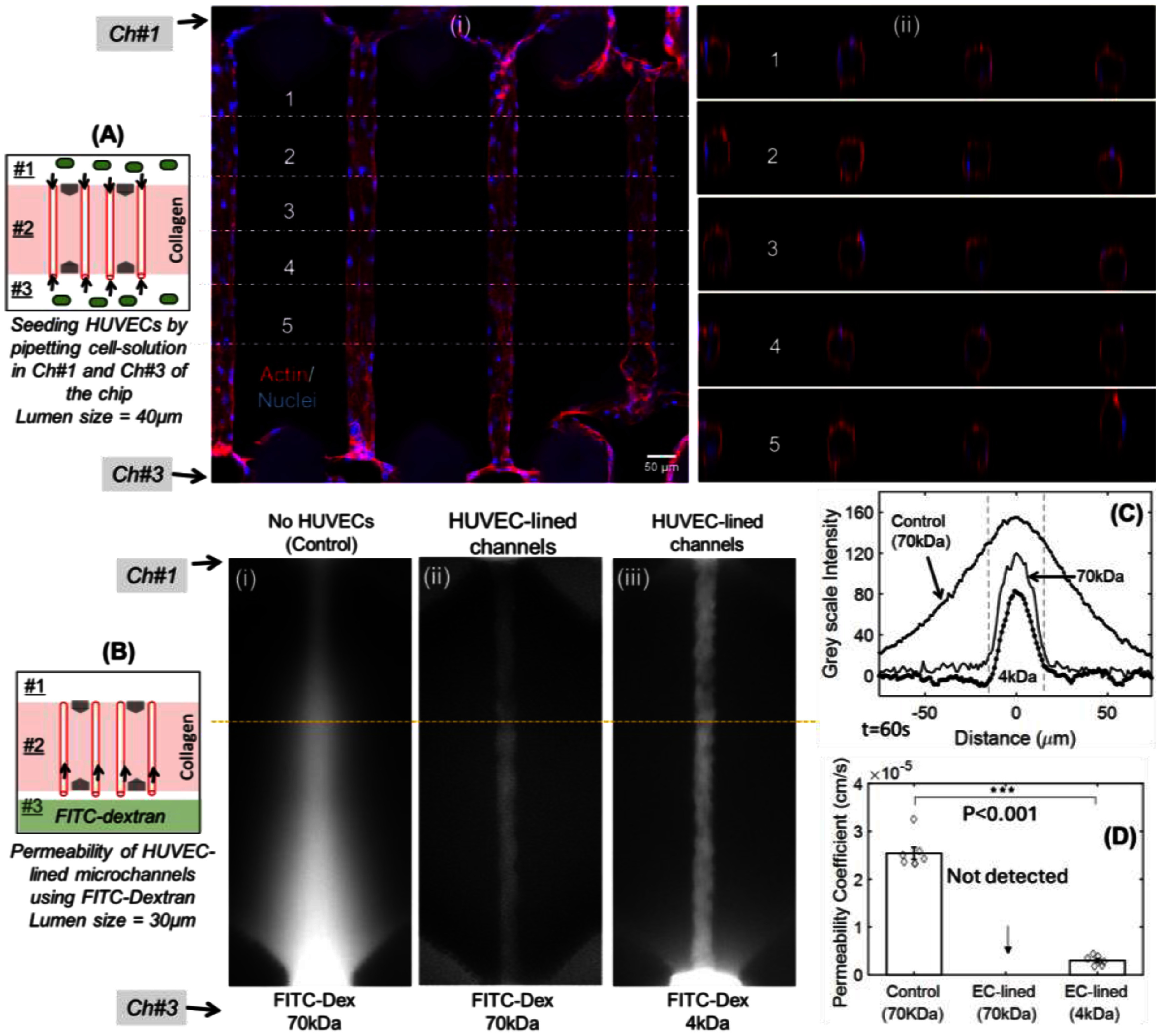
(A) (i) Schematic showing seeding of HUVECs in Ch#1 and Ch#3 of the chip to generate endothelialized microchannels (diameter = 40 *µ*m) embedded in collagen ∼50 *µ*m above the bottom glass coverslip. (i–ii) Representative fluorescence microscopy top and cross-sectional views highlighting cell morphology on Day 5. (B) Schematic showing the process flow for testing barrier function of artificial capillaries. (i–iii) Representative fluorescence microscopy images showing diffusion of FITC-dextran from the microchannels into the collagen under various conditions. (C) Fluorescence intensity plots comparing leakage of dye from the microchannels, (D) Permeability coefficient of artificial capillaries.

Model vascular endothelial cells (HUVECs), cultured under standard conditions, were seeded in chambers 1 and 3 at a concentration of 0.5 M cells ml^−1^, and 24 h later, perfusion flow was initiated using 1 ml media in pipette tips. Brightfield images show that cells readily migrate within the channels within 24–48 h, and by Day 4, microchannel surfaces are completely covered by HUVECs. Sometimes, when a seeding concentration of 1 × 10^6^ cells ml^−1^ or higher is used, channels can be blocked (arrow, SI-5), which can be removed by simply reversing the media flow by adding media-filled pipette to the other side-channel ports. For seeding concentrations of 0.5 M ml^−1^ or less, no blockages were observed, and unidirectional media flow was performed. Morphology of cells, characterized by staining for f-actin, shows uniform coverage of cells within channels of lumen size 40 *µ*m (figure 3(A)). Cross-sectional images show that lumens are open throughout the length of the channels. To characterize the patency and barrier function of these channels, FITC-dextran (70kDa) solution was used in chips with microchannels lined with or without HUVECs (figures 3(B) and SI-6). Results show that HUVECs prevent diffusion of dye into the surrounding collagen matrix. In fact, since the permeability for HUVEC-lined channels was undetectable with 70kDa dye, we repeated this experiment with a lower-molecular-weight FITC-Dextran dye (4kDa). Even the permeability coefficient for 4kDa (0.3 × 10^−5^ cm s^−1^, V:1) was found to be significantly lower than 2.54 × 10^−5^ cm s^−1^ obtained for control collagen channels (with 70kDa dye, V:2). This shows that endothelialized channels have robust barrier function. Here, we choose a lumen size of 30 *µ*m, and permeability was characterized using 3 independent chips with six measurements per chip.

### 2.4. Characterization of endothelialized lumens

**Figure 4.**
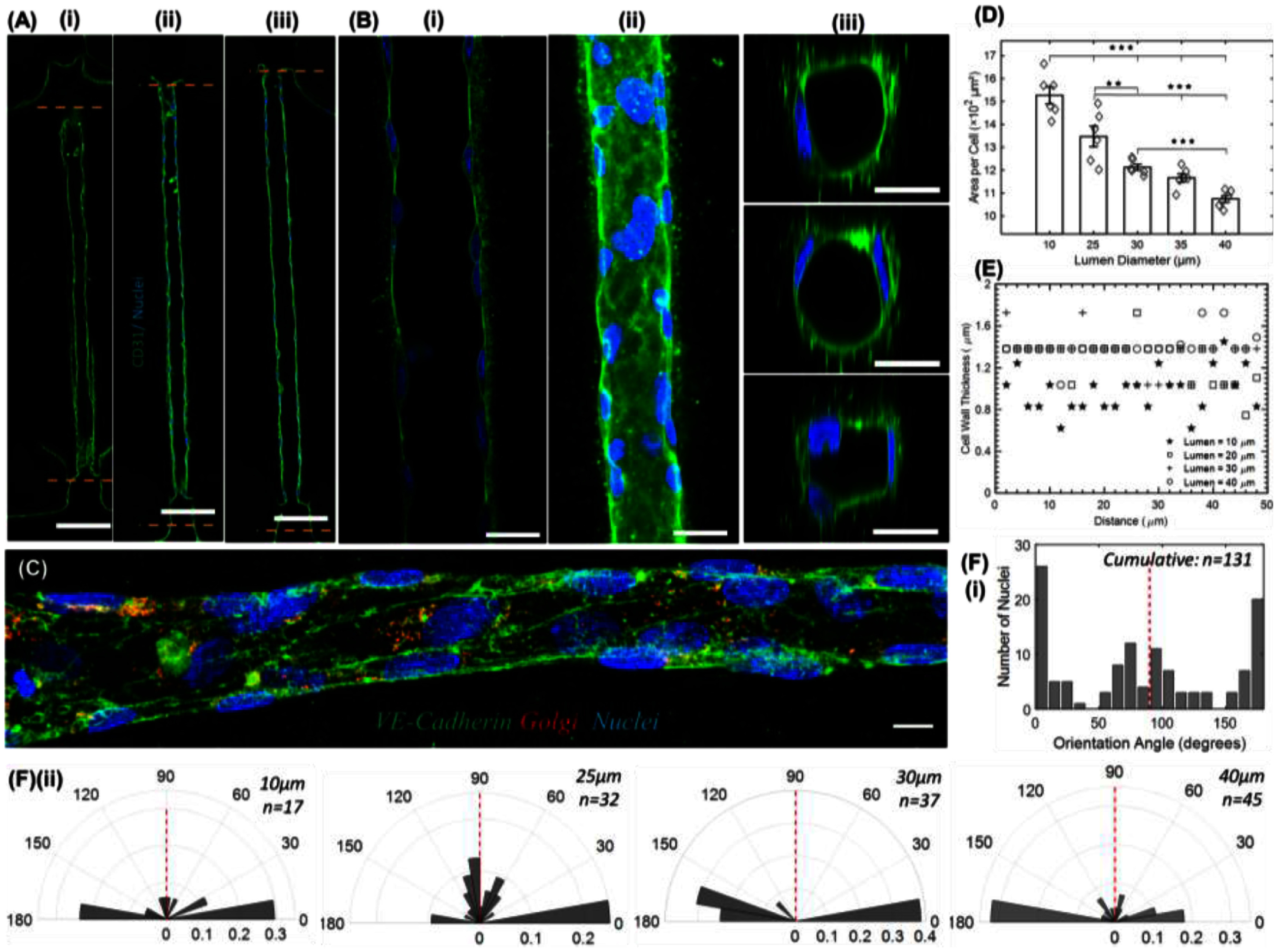
(A) Representative images showing (expression of CD-31 (PECAM-1, green). *φ* ∼ 25 *µ*m. (B) High resolution images at a single-plane (i), z-stack composite (ii), and cross-sectional images along the length of the microchannel (iii) (C) Images showing staining of VE-cadherin (green), Golgi apparatus (red), and nuclei (blue). Plots showing variations in cell area (D) and cell thickness (E) as a function of lumen size. (F) Plots showing changes in nuclei orientation with respect to flow direction. Scale bars: (A) 50 *µ*m, (B) 25 *µ*m, (C) 10 *µ*m.

Chips with HUVEC-lined microchannels were stained with CD-31, a marker for platelet/endothelial cell adhesion molecule-1 (PECAM-1), which is known to play a role in regulating vascular permeability. Fluorescence images show that artificial capillaries (*φ* ∼ 25 *µ*m) remain open and exhibit a monolayer of cells around the internal surfaces of the channels (figures 4(A) and (B), V:3,4) From the cross-section of endothelialized microchannels, we found that 1–3 nuclei were used to form the lumen based on the location within the microchannels (V:5–7). These structures resembled the cross-section of *in vivo* capillaries, with thin, elongated nuclei with slight bulges projecting into the lumen (figures 4(B-iii) and SI-7). We analyzed the changes in cell areas, thickness of the cytoplasm, and orientation of nuclei with reference to the direction of media flow (figure 4(D)–(F) and SI-8) Based on VE-cadherin staining, artificial capillaries exhibited an elongated cobblestone-like morphology, typically seen under unidirectional flow conditions (figure 4(C)). For Golgi apparatus, no polarization along the direction of flow was observed. Assuming complete coverage of HUVECs in the microchannels, based on our permeability results, we divided the total area of the cylindrical channel by the number of nuclei to get a metric of cell spread in channels with various lumen sizes. We found that, as lumen size decreases, cells spread more; fewer cells can spread and wrap around channels with high curvature (smaller lumen) (10 *µ*m vs 40 *µ*m) (figure 4(D)). The thickness of the cytoplasm for each lumen size was measured. Results show that for a lumen size of 10 *µ*m, a minimum thickness of 0.6 *µ*m was recorded, while a maximum of 1.8 *µ*m was calculated for larger lumen sizes (20–40 *µ*m) (figure 4(E)). Other than a lumen size of 25 *µ*m, all other lumen sizes show a nuclear orientation perpendicular to the direction of flow (figure 4(F)). Results in this section were obtained using 3 independent chips with six measurements per chip.

### 2.5. Customization of microchannel architecture

**Figure 5.**
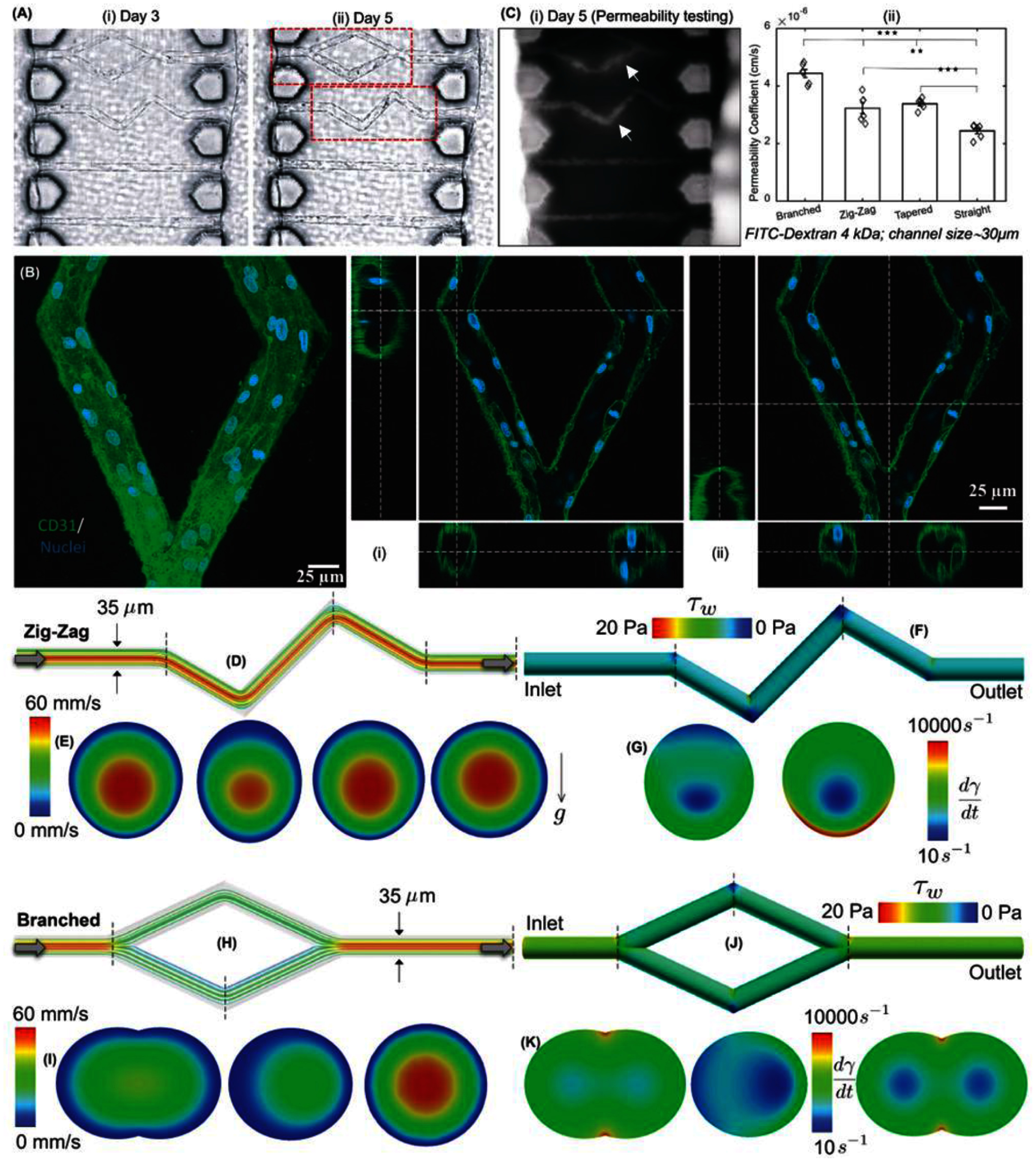
(A) Representative brightfield images showing artificial capillaries with custom geometries (branched, zig-zag, straight, tapered). (B) Cell morphology via PECAM staining within branched geometry capillaries. Lumen size = 30 *µ*m (C) Fluorescence image during permeability testing (i) and its quantification (ii). While arrows point to regional leaks in areas of high shear stresses. (D). Numerically simulated velocity streamlines in the zig-zag channel shape highlighted in panel 5Bii. (E) Cut-aways in the velocity field for the cross-sections marked by the dashed lines in panel (D), in the same left-to-right order. (F) Numerically simulated wall shear in the zig-zag channel. (G) Strain rate maps for the cross-sections marked by the dashed lines in panel (F), in the same left-to-right order. (H) Numerically simulated velocity streamlines in the branched channel shape highlighted in panel 5Bii. (I) Cut-aways in the velocity field for the cross-sections marked by the dashed lines in panel (H), in the same left-to-right order. (J) Numerically simulated wall shear in the branched channel. (K) Strain rate maps for the cross-sections marked by the dashed lines in panel (J), in the same left-to-right order.

To mimic the complexities of *in vivo* capillaries, we generated user-defined customized channel topologies followed by HUVEC seeding, as described earlier. Custom designs included, from the top (figures 5(A-i) and (ii)): (i) branched, (ii) zig-zag, (iii) tapered, and (iv) straight. Brightfield images show the formation of HUVEC-lined channels (figures 5(A-i), (ii) and SI-9). On Day 5, permeability was characterized. A cross-section of branched topology shows open lumen with nuclear morphologies similar to straight channel topology. (figure 5(B)) Permeability studies, performed on HUVEC-lined channels using FITC-Dextran (4kDa) on Day 5, show that the topology of the channels does play a role in their permeability properties (figure 5(C-i)). Some local regions of leakages can be seen in the branched and possibly the zig-zag lumen geometry, as opposed to straight lumens that show no leaks. Local permeability characterization shows that branched (4.5 × 10^−6^ cm s^−1^), zig-zag (3.237 × 10^−6^ cm s^−1^), and tapered (3.410 × 10^−6^ cm s^−1^) topologies show higher permeability as compared to straight lumen geometry (2.441 × 10^−6^ cm s^−1^) (figure 5(C)-(ii)). Additionally, the computationally simulated velocity fields (exemplified by 30 representative fluid streamlines) and the resulting area-weighted average of the wall shear for the two channel shapes highlighted in figure 5(B-ii) are included in figures 5(D)–(G) (for the zig-zag channel shape) and figures 5(H)–(K) (for the branched channel shape). The cross-sectional diameters of the in silico domains were maintained consistently at 35 *µ*m (in conformity with the physical design). Within the zig-zag channel, the four panels in figure 5(E) demonstrate the velocity fields at the cross-sections marked by dashed lines in figure 5(D), in the same left-to-right order. The two panels in figure 5(G) correspondingly show the strain rate contours within the bulk for the cross-sections marked by dashed lines in the wall shear demonstration in figure 5(F), in the same left-to right order. Similarly, within the branched channel, the three panels in figure 5(I) demonstrate the velocity fields at the cross-sections marked by dashed lines in figure 5(H), in the same left-to-right order. The panels in figure 5(K) show the strain rate contours in bulk for the cross-sections marked by dashed lines in figure 5(J)’s wall shear map, in the same left-to right order. As expected, the shear effects maximize at the corners of the channel geometries. The simulations are, however, representative, being only for the pressure head 5.3 cm (in this context, note the broader range of pressure heads tested in figure 2 in the simpler channel).

For the zig-zag channel, the simulation-derived mean outlet velocity and wall shear stress were 22.86 mm s^−1^ and 5.13 Pa, respectively. The overall range of wall shear (*τ_w_*) was 0–20.37 Pa, with the strain rates (d*γ*/dt) in the bulk ranging between 17.01 and 16 596.95 s^−1^. The latter could be used to compute the range of bulk shear *τ* = *µ*(dγ/dt), with the numerically implemented viscosity coefficient being *µ* = 0.001003 Pa.s (for water). Accordingly, in bulk, *τ* ∈ (0.017, 16.646) Pa. Next, for the branched channel, the corresponding estimates for the simulation-derived mean outlet velocity and wall shear stress were 31.63 mm s^−1^ and 4.98 Pa., respectively. Therein, the overall range of wall shear was 0–11.41 Pa, with the strain rates in the bulk ranging between 15.56 and 11 113.39 s^−1^. Following the same computation as above, the shear force in bulk turns out to be *τ* ∈ (0.016, 11.147) Pa.

To test whether engineered capillaries can be modified *in situ*, laser was used to remove collagen from target section to generate new microchannel connection between adjacent capillaries. Here, HUVEC-lined channels were generated in the chip, and on Day 5, a femtosecond laser was used to create connections between two HUVEC-lined microchannels. (SI-10, 11). Red arrows (SI-11(A)) show the presence of new microchannels that connect two adjacent HUVEC-lined microchannels, while red circle shows the generation of an incomplete channel, which is only open on one side. Brightfield snapshots show zoomed-in images of a liquid droplet flowing through the newly generated connection. (SI-11(B)) Within 24 h, cells migrate from the existing HUVEC-lined channels into the newly formed microchannel and generate a new branch in the network in 2–3 d. Confocal images show expression of PECAM while f-actin and nuclei morphology confirm the presence of open lumen and cell coverage within new branches (SI-11(C)). In one of the branches, marked by a circle, incomplete ablation of channel resulted in the creation of a dead-end. In this case, HUVECs migrated inside the channels but retracted soon after, resulting in a failed connection. In another experiment, GelMA (5%) was perfused into an engineered capillary, and an occlusion was generated by two-photon crosslinking; this decreases the lumen size from 20 *µ*m to ∼5 *µ*m (SI-12). Perfusion of FITC-dextran 2000kDa confirmed the presence of this partial occlusion, where flow can be seen to go around the partial occlusion.

### 2.6. Co-culture of model stromal cells with artificial capillaries embedded in collagen

**Figure 6.**
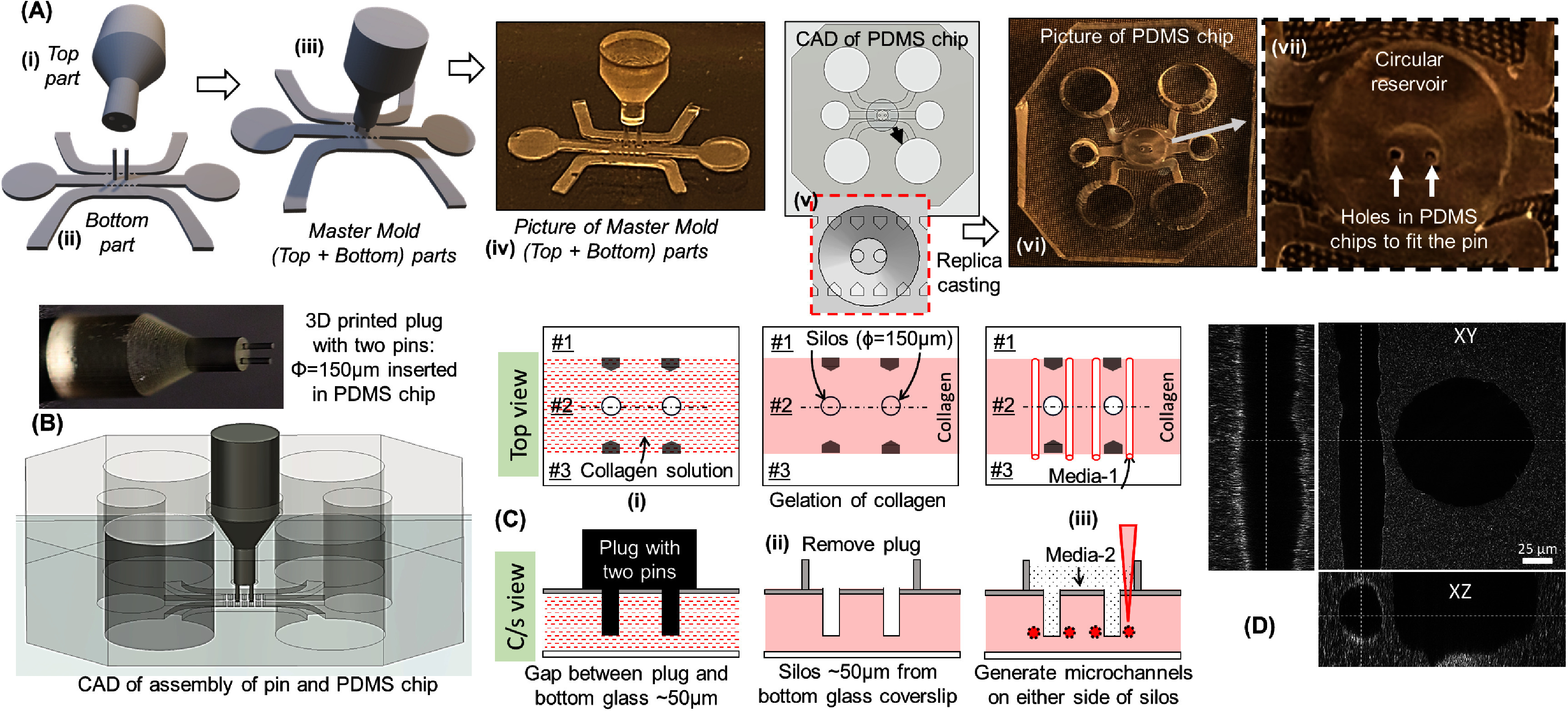
(A) Chip design B to facilitate co-culture experiments via simple cell-seeding. (A) Schematic showing the process flow: master mold is generated by assembling the top and bottom parts, then replica casting is used generate a three-chambered PDMS chip (iv)–(v) with a circular reservoir with two holes (vi)–(vii). (B) 3D printed plug (photography) with two pins inserted in PDMS chip (CAD assembly). (C) Schematic showing the process flow starting with perfusion of collagen solution in Ch#2, and post-gelation, pin is removed leaving behind silos. Then fs-laser cavitation is used to generate microchannels on either side of the silos. (D) Confocal reflectance microcopy images show the microchannel and silo.

To enable co-culture of endothelialized channels with model stromal cells (fibroblast-like 10T1/2s), we designed a new chip (design B). In this design, top and bottom parts, printed via PSLA, were assembled and replica casted using PDMS to fabricate three-chambered chips with a pair of silos in the central chamber (Ch#2). (figure 6(A) and SI-13–16, V:8). A conical plug with two pins (diameter = 150 *µ*m), printed using PSLA, was fitted into the central chamber of the PDMS chip, to ensure a vertical gap of ∼50 *µ*m between the base of the pins and the bottom coverslip (figures 6(B), SI-17 and 18, V:9). Then, rat-tail type I collagen solution (5 mg ml^−1^) was perfused in chamber #2 and allowed to gel at 37 °C for 30 min. Post gelation of collagen, the plug was removed to leave behind two cylindrical silos (∼150 *µ*m) embedded within gelled collagen in chamber 2 (figure 6(C)). The plug-pin design is such that a larger reservoir (∼3 mm, shown in figure 6(A-vii) was generated to enable easy pipetting of media and cell solutions. Femtosecond laser scanning was used to generate a pair of microchannels (∼30 *µ*m lumen size) on either side of the central silos. Reflectance confocal microscopy image shows both the central silo (∼150 *µ*m) alongside one of the microchannels at ∼50-100 *µ*m inside the gelled collagen matrix in chamber 2 of the chip (figure 6(D)).

**Figure 7.**
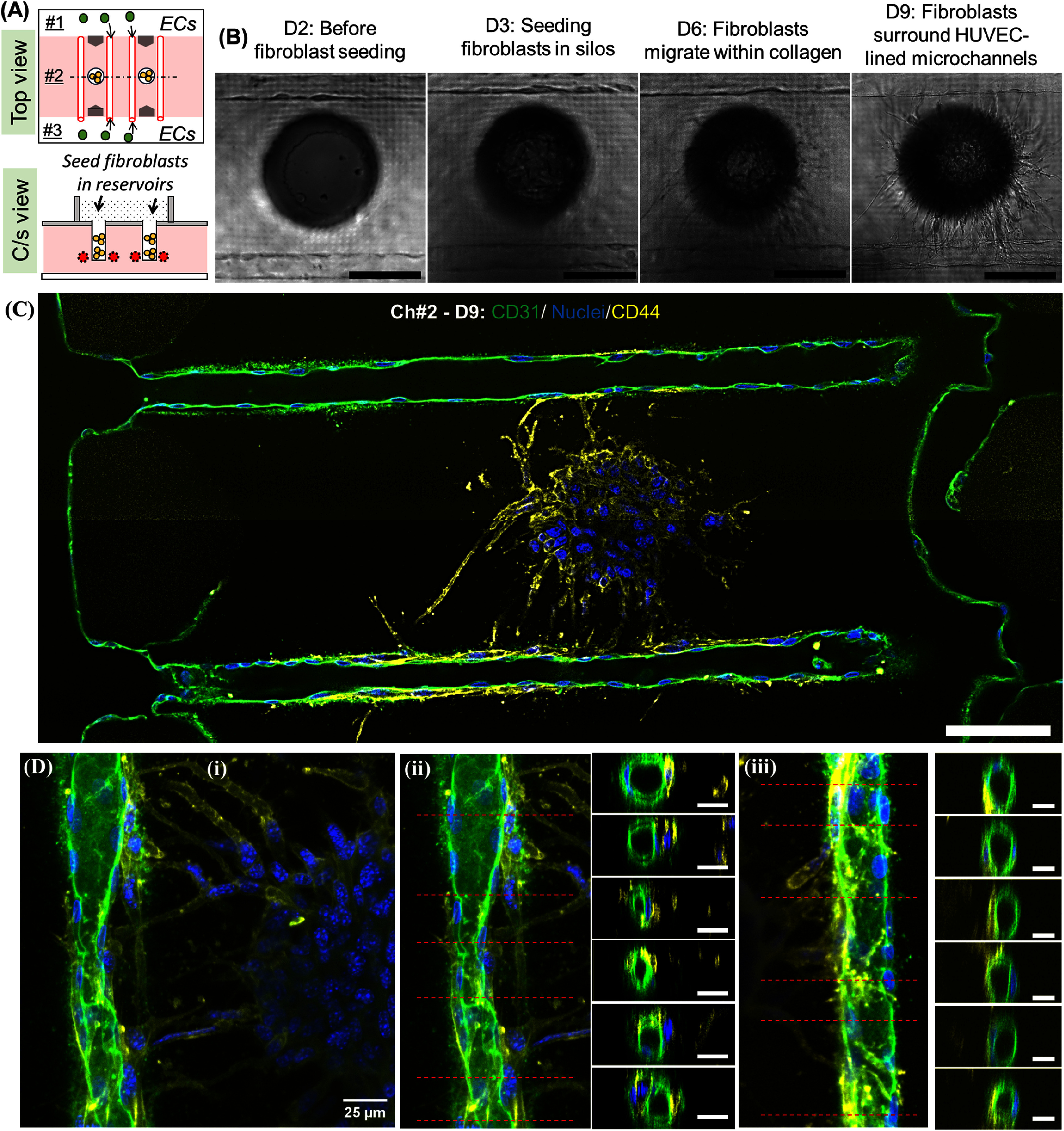
(A) Schematic showing co-culture of model stromal cells (fibroblasts-like 10T1/2s, gold color) in silos in close vicinity of HUVEC-lined microchannels (lumen size = 30 *µ*m) (B) Representative brightfield images showing addition of fibroblasts in silo on Day 3, followed by their migration into collagen matrix, and wrapping around artificial capillaries. (C) Titled confocal images showing the entire chip on Day 9. Image taken from the z-plane approximately at the center of the microchannels. (D) Confocal images of top and cross-sectional views from another chip showing stromal cell interactions with artificial capillaries (green). Scale bar B and C: 100 *μ*m; D: 25 *μ*m.

**Figure 8.**
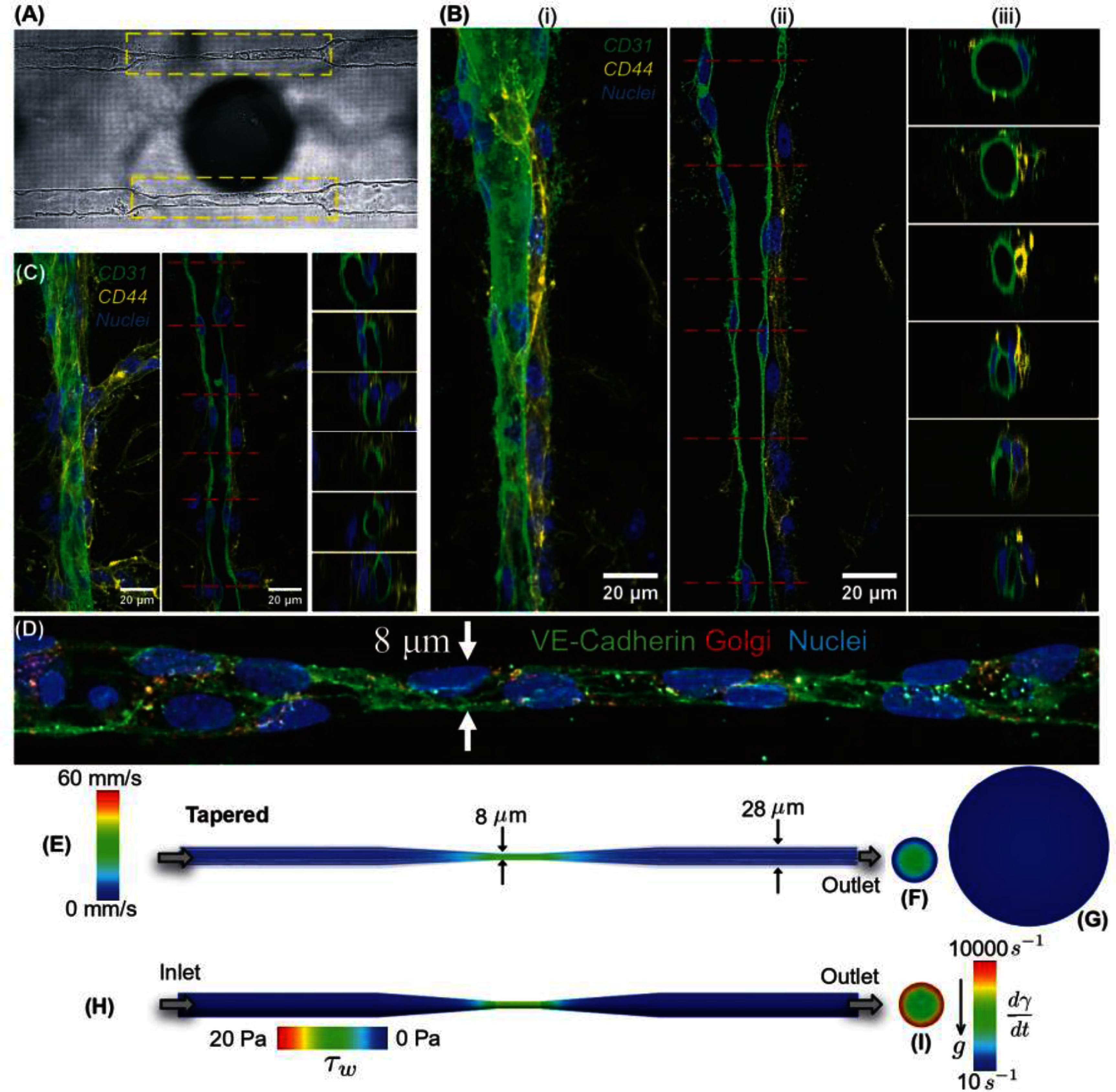
(A) Brightfield images of HUVEC-lined microchannels with change in lumen cross-section from 28 *µ*m to 8 *µ*m closely associated with a central silo. (B) and (C) Representative z-stacked, single-plane, and cross-sectional images showing fibroblasts (yellow) interacting with endothelialized microchannels (green). (D) Representative images showing staining of VE-cadherin (green), Golgi apparatus (red), and nuclei (blue). (E). Numerically simulated velocity streamlines for an idealized mid-tapered channel, mimicking the shape from panel (D). The inlet and outlet diameters in the computational domain were maintained at 28 *µ*m, while the diameter progressively decreased to 8 *µ*m at mid-section, (F)–(G). Cut-aways in the velocity field at the middle point of the channel and at the outlet, respectively. (H). Numerically simulated wall shear in the tapered channel. (I). Strain rate map at mid-section. Panels (F), (G) and (I) follow the same length scale.

HUVEC solution (0.5 M/cells) was pipetted in the side chambers of the chip to enable endothelialization in 2 d. On Day 3, model stromal cells (fibroblasts like 10T1/2s, 0.5 M/cells) were pipetted into the open reservoir on top of chamber 2. Brightfield images show migration of fibroblasts into the collagen matrix within 24 h, and they started to surround the endothelialized microchannels within 6 d figure 7(A) and SI-19. Tiled confocal images showing fibroblasts migrating from central silo (CD44, yellow) towards HUVEC-lined microchannels (CD31, green). By Day 6, fibroblasts are seen wrapping around the lumen from the outside (figures 7(B), SI-20, V:10,11). For some chips, we observe the fibroblasts migrate near the HUVEC-lined microchannels and actively decrease the lumen sizes (figure 7(C-i) and (ii)), while in some other chips, no change in lumen sizes was observed (figure 7(C-iii)) Next, we repeated the same experiments with lumen size of ∼8–12 *µ*m. Here, microchannels were generated such as their lumen size change cross-section from lumen size of 20 *µ*m to 8 *µ*m in the vicinity of the central silo (figure 8(A). While the lumen remains open, confocal images show that lumen size varies between 8 and 12 *µ*m as fibroblasts interact with HUVEC-lined channels (figures 8(B) and (C), V:12–15). VE-cadherin staining shows an elongated morphology possibly due to the ablated template as well as unidirectional flow conditions (figure 8(D)). We have also numerically simulated the transport trend in an in silico idealization of this channel shape. In this configuration, the inlet and outlet diameters were maintained at 28 *µ*m, while the diameter progressively decreased to 8 *µ*m at the mid-section. Under a simulated pressure head of 5.3 cm, the mean velocity at the outlet and the wall shear stress were estimated to be 0.95 mm s^−1^ and 0.84 Pa, respectively (see figure 8(E)). The cross-sectional views of the velocity fields are included in figure 8(F) (at the tapered center) and figure 8(G) (at the flow outlet). Figure 8(H) demonstrates the wall shear trend (maximizing at the tapered region), with strain rate fields in bulk shown in figure 8(I) (at the center). Herein, the maximum and minimum wall shear stress values are 17.857 and 0 Pa, respectively. The maximum and minimum strain rate values are 13 866.280 and 9.668 s^−1^. Panels F, G, and I are on the same scale. The co-culture experiment was performed using 3 independent chips for each lumen size.

## 3. Discussion

Capillaries, critical for nutrient exchanges between blood and surrounding tissues, can vary widely based on their location within the body. For instance, continuous capillaries (lumen size ∼5–10 *µ*m) are found in organs where selective exchanges are crucial (blood-brain barrier), while sinusoidal capillaries (lumen size ∼30–40 *µ*m), are found in organs that require the passage of larger molecules (liver, bone marrow). Moreover, drastic changes in capillary properties are often found in the same tissue. For instance, distinct capillary types were identified in long bones of mice [27–29] where oxygenated arterial blood is preferentially fed into type H columnar (straight) capillaries (∼10–15 *μ*m in diameter) followed by type L highly interconnected sinusoidal (branched) capillaries (∼20–30 *μ*m in diameter), before flowing into larger veins. Like capillary size and topology, their lengths could also vary widely between 50 and 500 *µ*m. Since capillary-stromal interactions play a vital role in tissue development, aging, and diseases, having the ability to reproducibly generate capillaries on a chip is vital for the discovery of unknown crosstalk mechanisms between capillaries and organotypic function.

At present, the ‘gold standard’ to make a capillary on a chip relies on self-assembly of vascular endothelial cells within relevant matrix (fibrin, collagen) [30–32], which results in randomly organized microvasculature with wide variations in lumen sizes, architectures, perfusability, and associated shear stresses. To address these issues, a wide range of *in vitro* models have been developed to study microvasculature biology. Most common models cast ECM gels around sacrificial molds (needles, stamps, patterns) to generate microvessels of defined sizes [3, 33, 34]. Despite its wide use in the field, capillary-sized lumens and complex topologies remain challenging with this method. Advances in extrusion bioprinting have made it possible to generate 3D channel topologies using a wide range of printable sacrificial materials [35–37]. Other methods, like electrohydrodynamic inkjet bioprinting can achieve a lumen size of 30 *µ*m [38], although low-viscosity bioinks result in less stable structures. Manual removal of melt electro-written PCL fibers from cell-laden hydrogels can generate lumen sizes from 10–41 *μ*m [39], although branched or curved topologies cannot be realized. Light-based methods such as laser tweezers, stereolithography, digital light processing, and volumetric bioprinting have also been developed to generate complex microvascular structures [40–44], although only multi-photon laser processing is capable of generating capillary-sized channels [45–50]. Moreover, challenges of media perfusion and endothelialization within capillary-sized lumens and incorporation of supporting cells in relevant ECM around the lumens remain to be solved. In this context, we report a new technology to generate artificial capillaries on a chip with custom control over lumen sizes and architectures using a combination of femtosecond laser cavitation and collagen casting within multi-chambered microfluidic chips.

We show that femtosecond laser-assisted cavitation can generate microchannels embedded within natural collagen matrix with defined lumen size range (8–40 *µ*m). We do not see any contraction of collagen channels after seeding HUVECs, as reported by other groups [6, 50], possibly due to a small cross-section of the microchannels and higher collagen concentration (5 mg ml^−1^) used in this work. We found that collagen below 2.5 mg ml^−1^ results in unstable microchannels, implying a stability threshold is necessary to generate structurally robust channels. Since the mechanisms governing laser-collagen interactions to generate a specific lumen size are still not completely known, we relied on trial-and-error to optimize the lumen sizes in this work. Although we focused on making in-plane microchannels ∼50–100 *µ*m inside collagen matrix, 3D out-of-plane channels are also possible by programming the stage accordingly, as shown in our previous work [51]. Processing of collagen within PDMS chips made using 3D printed molds offers fast processing times and design flexibility. In the future, the processing time can be substantially decreased by using a galvanometric scanner instead of a scanning stage used in this work. We chose easy-to-culture, inexpensive, and widely used HUVECs, as a model cell to allow direct comparisons with existing model systems, although our method can be agnostic of both ECM and cell types. To demonstrate this capability, we synthesized hyaluronic acid methacrylate (HAMA) hydrogel and photo-crosslinked it within the central chamber of the chip followed by laser ablation of microchannels of varying lumen sizes (3–40 *µ*m) (SI-21) Unlike protein-based collagen, hyaluronic acid is sugar-based component found in ECM. Next, we replaced HUVECs with endothelial colony forming cells (ECFCs) and show that engineered capillaries can be generated with no change in our protocol. ECFC are specific type of endothelial progenitor cell with a high proliferative capacity to form new blood vessels *in vivo*, and more importantly they are clinically accessible source of autologous ECs [52] (SI-22).

In this work, direct seeding of HUVECs in side-chambers for 24-hours followed by gravity-based perfusion culture for 48 h resulted in robust capillary-sized vessels in 3 d with robust barrier function up to 12 d. This is in contrast to reports that require a larger parent vessel before small-sized vessels can be grown [46]. We found that HUVECs preferentially align within the lumens rather than migrating into the surrounding matrix, similar to what others have observed in other hydrogel systems [53]. Since our longest duration was only 12 d, more work is needed to test the influence of lumen size and curvature, fluid flow type (static, unidirectional, bi-directional), and the functional expression of key biomarkers over longer culture durations. In our work, for lumen sizes (∼30 *µ*m), the lengths were the width of the chamber 2 (∼800 *µ*m)—an aspect ratio of ∼23, while for the smallest lumen sizes (∼8 *µ*m), the aspect ratio was ∼20. Further work is needed to study the effect of the aspect ratio of artificial vessels and their long-term stability. The barrier integrity was initially tested with 70 kDa FITC-Dextran (close to albumin in terms of molecular weight, roughly a physical size of ∼100 nm). Since the permeability coefficient for HUVEC-lined lumen was undetectable, we switched to a smaller size dye (4kDa FITC-dextran; similar to the size of a small protein or peptide, ∼1.5 nm), and even this showed a significant drop in permeability as compared to the controls that were performed with 70kDa dye. Our values are within the range reported in the literature for *in vitro* HUVEC micro-vasculature models and close to *in vivo* microvessels [54, 55]. We found that when the lumen size decreased, less number of HUVECs spread more within the lumen; this is similar to other reports [56], which used large-scale volume electron microscopy data from mouse cortex to show that microvessels in transitional zones near penetrating arterioles and ascending venules are larger and typically composed of 2–6 endothelial cells, while capillaries intervening these regions are constructed from either 1 or 2 endothelial cells. We characterized the endothelial vessel wall and observed nuclei protruding into the luminal space, similar to *in vivo* reports [56]. This work also showed the capability of *in situ* modification to existing artificial capillaries. Using two-photon crosslinking, we also show that user-defined obstructions can be added within the microchannels. Although these studies were conducted as proof-of-concept experiments, this strategy can be potentially used to study how user-defined editing relates to local tissue hypoxia, inflammation, and cell death, and whether blockages can be naturally resolved (recanalization) [57].

Since long-term stability of capillaries typically requires co-culture with supporting cells [58], it is important to show that support cells can be added at any point during active cell culture. We choose fibroblasts-like 10T1/2s as our model support cells due to their wide use in microvascular cocultures [59]. We designed a new chip with top-open silo arrays (diameter = 150 *µ*m) to facilitate seeding of relevant stromal cells during active cell culture. Since the timing of the addition of cells to a microvascular co-culture model has been shown to be critical [60], separate seeding ports in our chip provide control over seeding sequence/timing for each cell type during active cell culture. The depth of the silos is optimized such that floor of the silos are ∼50–100 *µ*m above the bottom glass interface; this ensures that all interactions between stromal cells (seeded in Ch#2) and artificial capillaries occur within natural 3D collagen matrix and facilitate high-resolution imaging. For PDMS chips, the time from design-to-use takes ∼28 h which is ideal to rapidly optimize device design. Collagen gelation takes 24 h while laser-assisted microchannel formation takes few hours; since the fabrication process to sculpt collagen gels via casting and laser cavitation are decoupled from the cell-seeding process, many chips can be fabricated, stored, and used when necessary.

To provide mechanistic biophysical insights related to artificial capillaries, the *in vivo* visualization of transport within the fabricated channels has been augmented with CFD simulations that relate the geometric signature of the artificial capillaries to quantitative, physiologically relevant metrics (namely, flow velocity, wall shear stress, and strain rates) that cannot be, otherwise fully resolved experimentally, due to the small lumen size range, custom architecture, and scattering due to the collagen matrix.

The CFD exercise captured the channel topologies and the precise intra-channel pressure heads from the experimental test cases, while generating the simulation-derived flow velocity and wall shear measures as predicted parameters. For validation of the numerical approach, the CFD-based velocity and shear values were compared and found to be in agreement with the corresponding experimental measurements from the straight channel (see figure 2). Although the flow regimes observed in the designed channels predominantly comprise laminar physics, the transport mechanism also encounters bifurcating spaces especially within the branched channels which may induce transitional flow behavior via recirculating shear layers. Consequently, we have employed LES modeling (a scheme for turbulent flow modeling, while the current system is largely laminar) since it acts effectively as a direct numerical simulation in regions where eddies are not present, with the sub-grid scale viscosity dropping to zero. The approach has enabled high-fidelity resolution of the transient, transitional, and potentially complex flow phenomena, with more accurate, non-stationary data. The superior accuracy does come with caveats of increased computing costs, though, warranting fine mesh resolution and small numerical time-steps. The scheme is, however, less expensive than a full-scale DNS. The LES-based gravity-driven simulation scheme was validated with bead-flow experimental measurements for the straight channel topology (figure 2, lumen size of 40 *µ*m) and predictive extrapolations were applied to the branched, zig-zag, and centrally tapered channel architectures. This model can enable the prediction of how the *in situ* architectural edits (in the form of, for example, new branches, occlusions, and tapering in SI-10, 11, and 12) and fibroblast-driven remodeling may redistribute shear and velocity fields, thereby linking structural tunability of the platform to mechanobiological cues that would be locally experienced by endothelial cells.

Table 1 in the supplementary section provides a systematic, head-to-head comparison of this works’ novelty as compared to two recently published papers related to laser-based microchannel fabrication methods. For most experiments, all laser processing of collagen is performed prior to seeding HUVECs or fibroblasts, thus avoiding direct exposure of lasers to cells. Although fs-laser ablation and associated cavitation have been shown to cause structural modifications in collagen and hydrogels [61, 62], we were not able to detect any collagen densification (figures 1(D) and SI-3). For *in situ* modification experiment (SI-11), laser scanning was used to generate a new channel between two HUVEC-lined channels. Although non-linear processing of fs-lasers at ∼800 nm is known to cause minimal thermal damage to the surrounding cells, we expect all the cells in the laser scanning path to be dead. Other studies have characterized the ‘cell-affected volume’ from fs-laser ablation to be within 10 *µ*m of the scanning paths, thus preserving the viability of the surrounding cells [49]. In our work, after new-channel ablation, we observed HUVECs from existing channels migrate into new channel branches within 24 h; this implies that despite endothelial disruption of cells in the laser scanning path, the cavitation process remains localized. We did not observe any local matrix damage, or damage to adjacent cells caused via phototoxicity and/or thermal stresses. The acellular chips exhibit excellent shelf-life, remaining stable for up to 3 months under hydrated storage conditions without deterioration, however, more work needs to be done to characterize functional stability of artificial capillaries over 12 d using physiologically relevant endothelial and perivascular cells. Variability in co-culture models, such as reduced lumen sizes of artificial capillaries by fibroblasts, potentially occur due to local differences in collagen density. Since this work relies on endpoint experiments, we possibly only capture one snapshot of the dynamic endothelial-fibroblast interactions occurring in the chip. Future experiments with live-cell fluorescent markers could provide new information on the real-time mechanics of capillary-stromal cell interactions. Our model provides independent control over lumen size (8–40 *µ*m), lumen geometry and associated flow properties, ECM composition (in this case, collagen, 5 mg ml^−1^, and HAMA), and seeding location and timing of stromal cells (fibroblasts-like 10T1/2s). We observed higher permeability in branched, zigzag, and tapered topologies compared to straight lumens, possibly due to disturbed flow dynamics, locally varying velocities and pressures, and associated changes in endothelial morphology and their nuclear orientation, often linked to endothelial dysfunction [63–66], and supported in the literature [67]. Computational modeling results from this work also point to variations in fluid velocities and shear stress in non-straight channel topologies. The ‘local regions of leakages’ observed in branched and zigzag designs likely occur in regions of high curvature, where cells must stretch further to maintain junctional integrity. Please note that these observations were made with model HUVECs and in the absence of pericytes, which are essential for microvascular homeostasis [68]. More work is needed to extend this model with tissue-specific endothelial cells and supporting cells to mimic physiologically relevant organ-specific microenvironments. The ability of these chips to be readily shipped to any lab and cultured with clinically relevant cell types and ECM compositions could enable the study of cross-talk between flow, varying based on location, geometry (curvatures), and lumen sizes ECM properties, and relevant perivascular cells necessary to maintain a stable and functional microvascular network [69–71].

## Data Availability

All data that support the findings of this study are included within the article (and any supplementary files). Supplementary data 1 available at https://doi.org/10.1088/1758-5090/ae7834/data1.

## Materials and methods

Please refer to the supplementary information (SI) section. This file also includes additional figures (S1 to S22), movie files (V1 to V17), and one Table and associated captions.

Videos can also be found at: https://github.com/psomanlab/Capillary-on-a-chip.
